# Speciation in the Deep Sea: Multi-Locus Analysis of Divergence and Gene Flow between Two Hybridizing Species of Hydrothermal Vent Mussels

**DOI:** 10.1371/journal.pone.0006485

**Published:** 2009-08-03

**Authors:** Baptiste Faure, Didier Jollivet, Arnaud Tanguy, François Bonhomme, Nicolas Bierne

**Affiliations:** 1 UPMC, Station Biologique de Roscoff, Roscoff, France; 2 CNRS UMR 7144, Station Biologique de Roscoff, Roscoff, France; 3 Université Montpellier II, Montpellier, France; 4 CNRS UMR 5554, Institut des Sciences de l'Evolution, Station Méditerranéenne de l'environnement littoral, Sète, France; Duke University, United States of America

## Abstract

**Background:**

Reconstructing the history of divergence and gene flow between closely-related organisms has long been a difficult task of evolutionary genetics. Recently, new approaches based on the coalescence theory have been developed to test the existence of gene flow during the process of divergence. The deep sea is a motivating place to apply these new approaches. Differentiation by adaptation can be driven by the heterogeneity of the hydrothermal environment while populations should not have been strongly perturbed by climatic oscillations, the main cause of geographic isolation at the surface.

**Methodology/Principal Finding:**

Samples of DNA sequences were obtained for seven nuclear loci and a mitochondrial locus in order to conduct a multi-locus analysis of divergence and gene flow between two closely related and hybridizing species of hydrothermal vent mussels, *Bathymodiolus azoricus* and *B. puteoserpentis*. The analysis revealed that (i) the two species have started to diverge approximately 0.760 million years ago, (ii) the *B. azoricus* population size was 2 to 5 time greater than the *B. puteoserpentis* and the ancestral population and (iii) gene flow between the two species occurred over the complete species range and was mainly asymmetric, at least for the chromosomal regions studied.

**Conclusions/Significance:**

A long history of gene flow has been detected between the two *Bathymodiolus* species. However, it proved very difficult to conclusively distinguish secondary introgression from ongoing parapatric differentiation. As powerful as coalescence approaches could be, we are left by the fact that natural populations often deviates from standard assumptions of the underlying model. A more direct observation of the history of recombination at one of the seven loci studied suggests an initial period of allopatric differentiation during which recombination was blocked between lineages. Even in the deep sea, geographic isolation may well be a crucial promoter of speciation.

## Introduction

Reconstructing the history of divergence and gene flow between closely-related organisms is a difficult task of evolutionary genetics that has recently received much attention [1]. The analysis of DNA sequence polymorphism at multiple loci associated with new statistical coalescence-based inferences now provides an elegant approach to test for the existence of gene flow during the divergence process [2]. This approach has been applied to a number of closely related species [3], [4]. Somewhat unanticipated, gene flow has been detected in most cases [5]. Taken at face value, these observations would suggest parapatric speciation as a more common mode of speciation than previously thought. However, it is not clear that the detection of gene flow necessarily means genes continuously exchanged during the whole divergence period. Secondary introgression can be difficult to separate from ongoing primary differentiation with gene flow (*i.e*. parapatry), because the high stochasticity of the coalescence process often leads to a full range of situations, from reciprocal monophyly to extensive allele sharing [6]. Furthermore, in both cases natural selection is expected to prevent gene flow in regions of the genome where species-specific adaptations operate while gene flow can still be substantial in regions lacking isolation genes, resulting in so-called semi-permeable barrier to gene flow [7].

We here address the issue of the history of gene flow during differentiation in an original environment, hydrothermal vents in the deep ocean. Oceanic ridges are motivating places to study mechanisms by which speciation occurs. Ridges are nearly all connected into a one-dimensional network that has spread over the globe following plate tectonics. Contrary to what prevails in surface, quaternary climatic oscillations should not have perturbed so much the deep ocean and it is unclear whether long episodes of geographic isolation could have occurred in the recent history of species inhabiting ridges. In addition, the high variability of hydrothermal vent conditions (steep thermal gradients and chemistry heterogeneity) could induce perfect conditions for ecological speciation. For example, because of the ultramafic nature of the oceanic crust beneath the Mid-Atlantic Ridge [8], hydrothermal vent emissions from the deepest sites are naturally enriched in methane but nearly depleted in sulphides whereas it is the reverse in the shallower sites [9]. Depth, hydrothermal fluid composition in heavy metals, sulphides and methane, temperature and symbiotic associations are highly fluctuant parameters, which are likely to induce local adaptation and possibly ecological speciation. On the other hand, the subduction of ancient rifts and the emergence of new ones might have led to either isolation of vent fields at a large spatial scale on the long term or to transient events of spatial isolation on the short term, both phenomena being thought to have deeply affected the hydrothermal vent fauna [10], [11]. The constant modification of the hydrothermal vent landscape due to the fluid displacement led to recurrent ‘crash and flush’ in populations that might likely promote speciation and cause species crypticism [12], [13].

Mussels from the genus *Bathymodiolus* [14] are one of the most widespread mollusc dominating deep-sea hydrothermal vents and cold-seeps communities throughout the world. Interestingly, *Bathymodiolus* mussels still possess a dispersive larval stage, a biological feature that has long questioned the possibility of geographic isolation in marine species [15]. The genus *Bathymodiolus* has diversified a few millions of years ago following an explosive radiation leading to a large number of geographic species [16], [17]. Two distinct species of *Bathymodiolus*, *B. azoricus* and *B. puteoserpentis*, were morphologically described from the northern part of the Mid-Atlantic Ridge. Previous studies using both rDNA ITS2 intergenic spacer [18] and a combination of mitochondrial DNA sequences and allozymes [19] demonstrated the hybridizing potential of these two species on a very restricted area where the distribution of the two species meet. Concordant narrow clines in allele frequencies have been described [19] together with strong cytonuclear disequilibria in intermediate populations [20]. The history of divergence and gene flow, however, remains to be settled. Indeed, the hydrothermal vent complex of *Bathymodiolus* species in the northern Atlantic extends over more than 3000 km along the ridge axis, with fields separated by large transform faults, which offset the rift over tens to hundreds kilometres [21], [22]. The distribution of *B. azoricus* is limited to the north because of the rise of the rift up to Iceland and its southern limit is the hybrid zone centred on the Broken Spur site (29°N). Conversely, the northern range limit of *B. puteoserpentis* is the hybrid zone while the southern limit is unknown. *Bathymodiolus* phylogeny clearly indicates that *B. azoricus* and *B. puteoserpentis* are sister species of the *Bathymodiolus boomerang* species complex that inhabit the deep Atlantic cold seeps situated both on the American and African margins [17]. Won et al. [20] therefore proposed that the Broken Spur vent field (29°N) hybrid population corresponds to the dual arrival of migrants from more extreme source populations. Actually, this situation may suggest that either (i) *B. azoricus* and *B. puteoserpentis* have recently colonised the Mid-Atlantic Ridge *via* two independent entrance points and subsequently met again to hybridise in a secondary contact zone, or (ii) that ongoing speciation is occurring between the two species, in which case one species would derive from the other along an environmental gradient (parapatric speciation).

DNA sequence polymorphism data from one mitochondrial and seven nuclear loci were here gathered in order to assess genetic diversity, population subdivision and gene flow within and between both Atlantic vent species, and to perform divergence/polymorphism analyses using the outgroup species *Bathymodiolus thermophilus* (Pacific Ocean). The goals of this study were: (1) to infer the existence of gene flow in the process of divergence by using the Isolation with Migration (IM) model [23], (2) to estimate the genome-wide level of gene flow and attempt to analyse its possible variation among genes, and (3) to determine whether genetic variation follows the neutral mutation-drift expectations.

## Materials and Methods

### Data collection

**Figure 1:**
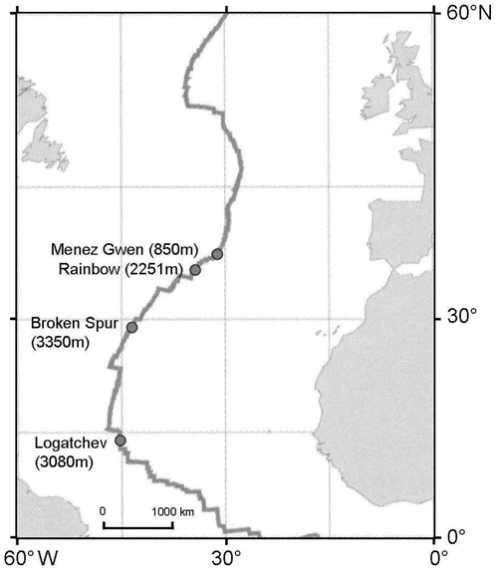
Position of Mid-Atlantic Ridge hydrothermal vent fields.

The three *Bathymodiolus* species were collected during the oceanographic cruises RV Sonne SO 157 2001, ATOS 2001, BioSpeedo 2004 (both on board the RV L'Atalante), Momareto 2006 and Serpentine 2007 (both on board of the RV Le Pourquoi Pas?). The first species, *Bathymodiolus azoricus*, was sampled from the sites Rainbow (2500 m; 36°13′N–33°54′W) and Menez Gwen (850 m, 37°50′N–31°31′W) at the Azorean triple junction. The second species, *Bathymodiolus puteoserpentis*, was sampled at the site Logatchev (3080 m; 14°45′N–44°58′W) on the Mid-Atlantic Ridge (MAR) (Figure 1). These two first species were collected using the telemanipulated arm of the ROV Victor6000. The third species, *Bathymodiolus thermophilus*, was sampled at 7°25S vent field (2450 m; 7°25′S–107°47′W) on the East-Pacific Rise (EPR) using the telemanipulated arm of the manned submersible Nautile, and on the Foundation hot spot (37°S on the Pacific Antarctic Ridge) (2200 m, 37°70S–110°87′W) using a dredge and a chain bag. Samples were brought back to the surface alive, subsequently dissected on board. Foot tissue and gills were immediately frozen and kept in liquid nitrogen until nucleic acid extraction back in our laboratory. DNA was extracted using the classical phenol-chloroform protocol [24]. A total of 44 and 52 individuals were used for the Rainbow and Menez Gwen *B. azoricus* populations respectively, 33 individuals for the Logatchev population of *B. puteoserpentis*, and 4 and 2 individuals for the outgroup *B. thermophilus* at the 7°S sites and 37°S respectively.

### PCR amplification, cloning and sequencing

**Figure 2:**
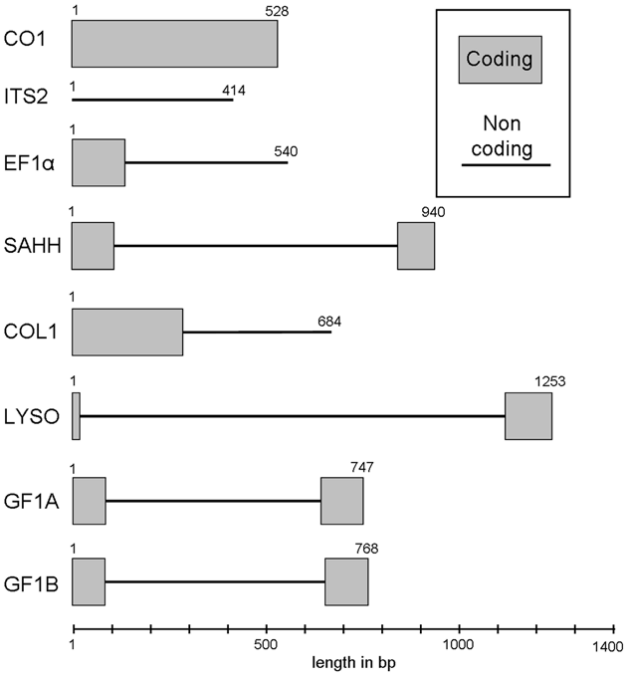
Size and constitution of the eight gene fragments studied.

DNA sequences were obtained for 7 nuclear loci and one mitochondrial gene (Figure 2). Six are protein coding genes, Elongation Factor 1α (EF1α) (FJ767126 to FJ767244); S-adenosylhomocysteine hydrolase (SAHH) (FJ767612 to FJ767709); Collagen type XIV (COL1) (FJ766982 to FJ767125), Lysozyme (LYZO) (FJ767466 to FJ767611), Ferritin GF1 (with two distinct paralogues: GF1A and GF1B) (FJ767245 to FJ767353 and FJ767354 to FJ767407). The two other loci are the mitochondrial Cytochrome-*C* Oxydase subunit 1 (CO1) (FJ766849 to FJ766981) and the ribosomal internal spacer 2 (ITS2) (FJ767408 to FJ767465). Targeted nuclear genes were obtained from a collection of *B. azoricus* ESTs (full-length cDNA library, [25]).

PCR amplifications were done with specific sets of primers designed from the conserved exonic regions of *B. azoricus* EST sequences, for which an intron was known to occur in other taxa. The sequences of the primer sets are summarised in Table 1. Primer sets and conditions used for CO1, EF1α and for Lysozyme have been described elsewhere [26]. For new loci, genes were amplified and cloned following the Mark-Recapture (MR) method [27] (see also [26], [28]) with 4-nucleotides 5′-tailed primers (4 bp polynucleotides tag at their 5′ ends). Reactions were performed in a 20 µl volume containing 1X PCR buffer (supplied with polymerase enzyme), 2 nM MgCl_2_, 0.25 µM of each dNTPs, 0.4 µM of each primer, 0.025 U of Thermoprime Plus *Taq* polymerase (ABgene), and 20 ng genomic DNA. Cycling parameters were 94°C for 5 min, 35 cycles of 94°C for 45 s, 60°C for 1 min, 72°C for 2 min; and a final elongation at 72°C for 10 min for each gene. Every individual was PCR-amplified separately with different sets of 5′-tagged primers. Because we used MR cloning protocol, PCR products were mixed together (equimolar) and cloned into a pGEM-T vector by using the pGEM-T cloning kit (Promega, Madison, WI, USA). We used a capture effort of two in MR-cloning: 96 clones per population and per gene were sequenced at the Genoscope (Evry) (http://www.genoscope.cns.fr/) with the universal primer sp6 or T7 flanking the insert on the plasmid. A total of 4300 initial sequences was used for this study (direct sequencing for CO1 in both strands, and 96 clones sequenced in both strands, for 3 populations for the 7 nuclear genes and the outgroup).

**Table 1:** Sequences of PCR primers.

locus	5′-[size of the tag] primer Forward -3′	5′-[size of the tag] Primer Reverse -3′
CO1	ATA AAA AGA TGT ATT RAA RTG ACG	TGT GGT CTG GAA TAA TTG GAA
EF1α	[3bp] ACG CCT GGG TAT TGG ACA AAC T	[3bp] CCA AGA GGG GTC GTA CAA ATT A
SAHH	[4bp] TAA ATC TTG GTT GTG CTC ATG GTC ATC CA	[4bp] TTT GAA TGG TCC TTC TTT AGG TAG AC
COL1	[4bp] TTA CCA AAT CAC AGG TCG GGG TCA TG	[4bp] TCT CCT TCG CTA TTT TTG TGG G
LYSO	[4bp] GTT TCC CCA AAA TGT ATG AGC TGT	[4bp] TAA TCT CCG CCT GGA CTA CCA CAA TC
GF1A and GF1B	[4bp] ATG TCT CAA AGT CAA CCT CGT CAA AAT	[4bp] TTG ATG TCC TGA AGA ACG ATT CGT CCT CC
ITS2	ACA TTG CGG CTT TGG GTC AC	GCT TAA ATT CAG CGG GTA CT

### Data analyses

For all genes, sequences were checked and edited using the Chromas 2.22 computer program (Technelysium Pty. Ltd., Helensvale, Australia). The sequences from each dataset were initially aligned with the program ClustalW [29] in BioEdit Sequence Alignment Editor [30], and improved manually. Artefactual mutations and *in vitro* recombinants were discarded when detected from alleles sequenced several times in multiple-recaptures. In order to prevent from sampling bias, only one sequence per individual was kept. In the specific case of heterozygote individuals, when both allelic forms were captured, the most frequently captured allele was retained. We used the two populations of *B. azoricus* as distinct samples (never pooled) in order to test whether a “site effect” occurred between Menez Gwen (850 m) and Rainbow (2500 m) and to get partial replicates when comparing *B. azoricus* and *B. puteoserpentis*.

As intragenic recombination was often detected, gene trees do not necessarily reflect gene genealogies. To represent an overview of the genetic relationships among alleles we inferred a network with the NeighborNet algorithm [31] in the software SplitsTree4 [32]. In addition, we paid a special attention to reconstructing gene genealogies for two loci which hold useful information. For the SAHH locus, recombination was not detected and the single possible gene tree was reconstructed with Genetree [33]. For the GF1B locus, in which a few recombination breakpoints and a few recombinant alleles were easily identified, we reconstructed the more parsimonious genealogy without recombinants and added recombinants afterwards with the help of the ancestral recombination graph reconstruction made possible with the Beagle software [34].

For each gene, the number of segregating sites (*S*), nucleotidic diversities (θ_w_ [35] and θ_π_ [36]), Tajima's D statistic [37], Ramos-Onsins and Rozas's *R_2_* statistic [38] minimum number of recombination events (Rm), [39] and absolute divergences (D_XY_) were estimated using the DnaSP version 4.10 software package [40]. Genetic differentiation between populations was estimated with the φst statistic analysis carried out with the Arlequin software [41], using a matrix of Tamura-Nei's distances between individuals; with 1000 permutations. Statistical significance of Tajima's *D* tests for each locus and sample was assessed by coalescent simulations with the software HKA (J. Hey's web page: http://lifesci.rutgers.edu/~heylab/HeylabSoftware.htm#HKA). Although coalescence simulations are conditioned on Theta and not on the number of segregating sites, they are based on the HKA protocol [42], a multilocus framework which incorporates the information contained in the divergence with the outgroup (*e.g*. mutation rate). We nonetheless verified whether the results were different when conditioning simulations on S, and they were not. According to Ramirez-Soriano *et al.* [43], the *R_2_* statistic was tested by coalescence simulations conditioned on S. A multilocus version of the HKA test [42], a neutrality test that compares divergence and polymorphisms at several loci, was performed on the 8 loci with the HKA software in order to test whether differences in tree topologies reflect differing levels of evolutionary rates or selective processes. In this latter test, a locus under positive selection is supposed to have a polymorphism/divergence ratio smaller than that of a neutral one. Finally, when possible we also used the coalescence-based maximum-likelihood method of Galtier, Depaulis, and Barton [44]. This method is designed to detect a distortion in the shape of gene genealogies generated by a diversity-reducing event (hitchhiking or bottleneck). The likelihood of a model in which a drop in effective size of strength S occurred at time T in the past is compared to the likelihood of a constant-size model.

### Fitting an Isolation-with-Migration model

To discriminate between the relative effects of divergence and gene flow on the speciation process, we analysed our data set under the Isolation with Migration model [45]. The model assumes that an ancestral population splits into two descendant populations that may continue to exchange genes after separation. The method estimates posterior probability distributions for both ancestral and actual population sizes, directional migration rates between the two populations, and the time elapsed since population splitting. A MCMC approach is used to draw a sample from the posterior distribution of genealogies and to estimate population parameters. The posterior distributions of migration rates and population sizes are derived analytically from the sampled genealogies [46], [23]. Convergence by the Markov chain simulations toward the true stationary distribution is checked by monitoring multiple independent chains started at different starting points and by assessing the autocorrelation of the parameter values over the course of the run. We also used the procedure for swapping among the cold primary chain and multiple heated chains (Metropolis coupling) to further ensure that the distributions we obtained actually reflected the stationary distributions [47]. Each locus was assigned an inheritance scalar, to adjust for its relative expected effective population size: 1.0 for autosomal locus, 0.25 for mtDNA. The method assumes no recombination and allows two mutation models: infinite sites (IS) and HKY with back mutation [48]. We chosed the HKY model and used the largest fragment without recombination for the analyses.

We first conducted a multi-locus analysis with the IMa program [45] and secondly analysed the migration rates of each gene separately with both the IMa and the IM program [46], [23]. Using the IM program allowed us a parameterisation of individual migration rates for different loci while theta and divergence time remained multi-locus estimates. The 90% highest posterior density (HPD) interval, that is the shortest span that includes 90% of the probability density, was recorded as. Conversion into effective population size estimates was done from the relationship N_e_ = θ/4*u*. Migration parameters of the model were used to calculate the number of migrants exchanged between the two hybridising species (i.e., M = 2*Nm*, the product of the effective number of gene copies and the per gene copy migration rate). Thus, M = 2*N_e_m* = (4N_e_ *u* × m/*u*)/2 = θ × m/2 [23].

When the method reveals nonzero migration rate estimates, the posterior distribution of the mean time of migration events was estimated for trees sampled from the Markov chain that had at least one migration event (given by the IM program) following Won and Hey [3].

## Results

### Genetic variation

To avoid a sampling bias in estimates and tests that assume random sampling, we used one multiple captured allele per individual, this allele being the most frequently captured in our mark-recapture cloning procedure [27]. From both Atlantic species and the outgroup *B. thermophilus*, a total of 559 sequences have been analysed at one mitochondrial locus and 7 nuclear loci, leading to a global number of 437 756 bases sequenced (745 bp per sequence in average). Allelic and nucleotide diversities as well as divergence to the outgroup are summarized in Table 2 for the three *Bathymodiolus* species. The weighted mean of Watterson's *θ* estimators [35] across autosomal loci (*i.e*. without CO1), were 0.0076, 0.009 and 0.0101 for *B. azoricus* (Rainbow), *B. azoricus* (Menez Gwen), *B. puteoserpentis*, respectively. The average divergence to the Pacific species *B. thermophilus* at autosomal loci was 3.6%. The divergence observed at the mtDNA locus was much higher (11.8%) while the diversity was lower than the nuclear average, in accordance with a higher rate of evolution and a smaller effective population size of the mitochondrial genome. Pairwise Φst estimates between the two *B. azoricus* populations were never significantly different from zero. As a consequence the two *azoricus* samples were pooled in following analyses. The between-species comparison (*B. azoricus*/*B. puteoserpentis*) detected significant differences at five loci: CO1 (*P* = 0.01), SAHH (*P* = 0.01), LYSO (*P* = 0.01), EF1α (*P* = 0.05), and ITS2 (*P* = 0.05).

**Table 2:** Summary statistics of nucleotidic polymorphism for mitochondrial and nuclear sequences.

locus	Species	*N*	*L*	*S*	θ	π	Tajima's D	*R_2_*	Div. (vs. 7S)	Div. Ba/Bp	Div. Rb/MG	Rm	Ф_st_	
													MG	Bp
CO1	*Ba*_Rb	44	528	26	0.0113	0.0058	−1.6176 *	0.051**	0.1181	0.0611	0.0048	4	0.0217	0.94***
	*Ba*_MG	52	528	18	0.0075	0.0036	−1.6411*	0.050*	0.1178	0.0609	SAA	4	-	0.96***
	*Bp*_Lg	33	528	3	0.0014	0.0006	−1.3758	0.069*	0.1190	-	-	0	-	-
	*Bt*_7S	4	528	0	0.0000	0.0000	-	-	-	-	-	-	-	-
EF1α	*Ba*_Rb	14	540	5	0.0030	0.0013	−1.8893*	0.144	0.0258	0.0019	0.0009	0	0.0090	0.08*
	*Ba*_MG	18	540	2	0.0011	0.0004	−1.5078*	0.157	0.0254	0.0014	SAA	0	-	0.09*
	*Bp*_Lg	15	540	6	0.0035	0.0021	−1.3326	0.107	0.0251	-	-	0	-	-
	*Bt*_7S	4	540	1	0.0010	0.0009	-	-	-	-	-	-	-	-
SAHH	*Ba*_Rb	13	940	18	0.0064	0.0048	−1.0545	0.102	0.0396	0.0134	0.0050	0	−0.0404	0.56***
	*Ba*_MG	13	940	22	0.0079	0.0055	−1.3315	0.083**	0.0405	0.0144	SAA	0	-	0.56***
	*Bp*_Lg	11	940	26	0.0101	0.0075	−1.1897	0.118	0.0384	-	-	2	-	-
	*Bt*_7S	4	940	14	0.0088	0.0100	-	-	-	-	-	-	-	-
COL1	*Ba*_Rb	18	683	37	0.0162	0.0116	−1.1564	0.082*	0.0405	0.0106	0.0110	2	−0.0160	0.05
	*Ba*_MG	31	683	42	0.0154	0.0109	−1.0809	0.073*	0.0407	0.0099	SAA	4	-	0.01
	*Bp*_Lg	24	683	32	0.0125	0.0087	−1.1690	0.076*	0.0408	-	-	1	-	-
	*Bt*_7S	1	683	/	/	/	-	-	-	-	-	-	-	-
LYSO	*Ba*_Rb	32	1297	58	0.0123	0.0120	−0.0418	0.115	0.0587	0.0141	0.0120	11	−0.0035	0.20***
	*Ba*_MG	35	1297	59	0.0129	0.0124	−0.1372	0.114	0.0581	0.0142	SAA	17		0.19***
	*Bp*_Lg	25	1297	50	0.0111	0.0101	−0.3588	0.115	0.0586	-	-	9	-	-
	*Bt*_7S	4	1297	16	0.0071	0.0068	-	-	-	-	-	-	-	-
GF1A	*Ba*_Rb	24	748	22	0.0079	0.0061	−0.8371	0.090	0.0213	0.0072	0.0070	2	0.0280	0.03
	*Ba*_MG	20	748	25	0.0098	0.0074	−0.9462	0.100	0.0229	0.0074	SAA	5	-	−0.03
	*Bp*_Lg	18	748	27	0.0105	0.0079	−1.0133	0.103	0.0228	-	-	5	-	-
	*Bt*_7S	4	748	9	0.0066	0.0065	-	-	-	-	-	-	-	-
GF1B	*Ba*_Rb	14	768	25	0.0106	0.0137	1.2203	0.197	0.0318	0.0152	0.0179	3	0.0591	0.02
	*Ba*_MG	13	768	39	0.0174	0.0201	0.6576	0.176	0.0316	0.0172	SAA	2	-	−0.06
	*Bp*_Lg	13	768	27	0.0117	0.0161	1.6281*	0.215*	0.0300	-	-	3	-	-
	*Bt*_7S	4	768	5	0.0036	0.0033	-	-	-	-	-	-	-	-
ITS2	*Ba*_Rb	14	413	6	0.0049	0.0030	−1.3944	0.178	0.0326	0.0082	0.0042	0	0.0231	0.05
	*Ba*_MG	14	413	13	0.0107	0.0053	−2.0740*	0.139	0.0318	0.0094	SAA	0	-	0.06*
	*Bp*_Lg	22	413	28	0.0210	0.0127	−1.5021	0.078*	0.0326	-	-	1	-	-
	*Bt*_7S	4	413	3	0.0041	0.0042	-	-	-	-	-	-	-	-

*Ba*: Bathymodiolus azoricus (Rb: Rainbow; MG: Menez Gwen); *Bp*_Lg: *B. puteoserpentis* Logatchev; *Bt*_7S: *B. thermophilus* 7°S.

N: Number of sequences.

L: Average length (bp) of the sequences from each species.

S: Number of polymorphic sites.

θ: Estimate of 2N_f_μ (*mtDNA*) (N_f_ is the effective number of females) or 4Neμ (nuclear DNA) per base pair using the number of polymorphic sites [35].

π: Estimate of 2Nfμ (mtDNA) or 4Neμ (nuclear DNA) using the average number of nucleotide difference per site [36].

Tajima's D: Tajima's statistic [37].

*R_2_*: Ramos-Onsins and Rozas statistic [38]. Significativity tested by coalescent simulation (given the number of segregating sites and no recombination) (*: p<0.05; **: p<0.01).

Div.: Average number of nucleotidic substitutions per site between populations. SAA: Same As Above.

Ф_st:_ population differentiation, tested by 1000 permutations with the Arlequin software (*: p<0.05; ***: p<0.001).

### Contrast of the phylogenetic signal across gene trees

**Figure 3:**
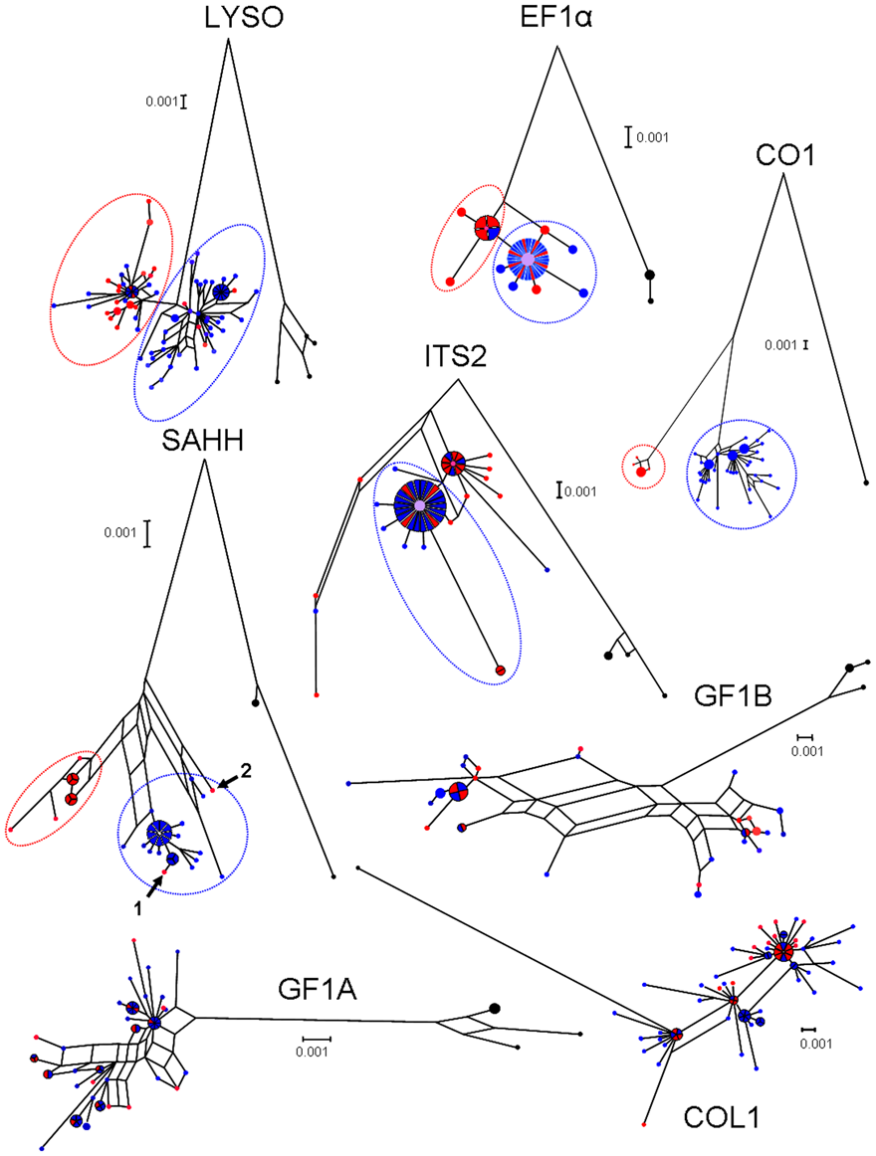
Networks inferred with the NeighborNet algorithm for the 8 loci. In red: alleles from *B. azoricus*; in blue: alleles from *B. puteoserpentis*. Small arrows: introgressed alleles (see discussion).

All loci displayed a large number of parsimony informative sites (77 per locus on average when excluding the outgroup). Networks inferred with the NeighborNet algorithm depict the genetic relationship among alleles for the 8 genes in Figure 3. The mitochondrial locus CO1 is the only one that displayed complete reciprocal monophyly of alleles sampled in both species. For the nuclear genes SAHH and LYSO, two well defined groups of alleles were observed, each of which preponderantly composed of genes sampled in the same species with only a few genes sampled in the other species. To a lesser extent, the ribosomal spacer ITS2 and EF1α tended to fit fine with the species dichotomy *B. azoricus* and *B. puteoserpentis*. The three other genes COL1, GF1A and GF1B displayed highly reticulated networks and an absence of association with geography (species). Although one could have attempted to define two groups of alleles at loci GF1A and GF1B, there was no possibility to attribute these groups to a given species. The position of the outgroup and the shape of the network obtained at locus COL1 did not produce phylogenetic relationships which easily fit with a long period of divergence between two gene pools.

The five loci which retained a probable footprint of differentiation were used to define two groups of alleles (ellipses in Figure 3) and the frequency of such defined synthetic alleles was reported in Table 3.

**Table 3:** Distribution of alleles.

		number of alleles per clade		proportion of alleles per clade	
		“Bazori”	“Bputeo”	“Bazori”	“Bputeo”
CO1	*B. azoricus_Rb*	44	0	100	0
	*B. azoricus_MG*	52	0	100	0
	*B. puteoserpentis*	0	33	0	100
SAHH	*B. azoricus_Rb*	13	0	100	0
	*B. azoricus_MG*	13	0	100	0
	*B. puteoserpentis*	2	9	18	82
LYSO	*B. azoricus_Rb*	24	8	75	25
	*B. azoricus_MG*	25	10	71	29
	*B. puteoserpentis*	5	20	20	80
ITS2	*B. azoricus_Rb*	10	4	71	29
	*B. azoricus_MG*	12	2	86	14
	*B. puteoserpentis*	8	14	36	64
EF1α	*B. azoricus_Rb*	13	1	93	7
	*B. azoricus_MG*	17	1	94	6
	*B. puteoserpentis*	9	6	60	40
GF1A	No differentiation and/or complex coalescence				
GF1B					
COL1					

**Figure 4:**
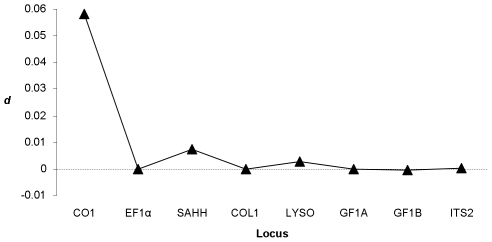
Difference in sequence divergence between *B. azoricus* and *B. puteoserpentis*. Each point represent the value of absolute divergence *d* for the locus shown on the x-axis: *d* = *d_azo/puteo_* − (π_puteo_+π_azo_)/2 where *d_azo/puteo_* is the mean interspecific sequence divergence between *B. azoricus* and *B. puteoserpentis*, and (π_puteo_+π_azo_)/2 is the mean population diversity between *B. azoricus* and *B. puteoserpentis*. Under a strict isolation model consistent with the traditional phylogeny (strict divergence *azoricus/puteoserpentis*), the value of *d* is expected to be greater from zero for most loci.

**Figure 5:**
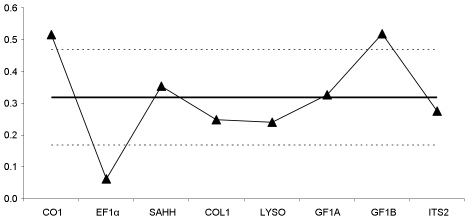
D_azori/puteo_/D_ATL/PAC_ ratio for the 8 loci studied. The horizontal line represents the average divergence between the Atlantic and the Pacific. Doted lines correspond to the standard deviation.

Variations in phylogenetic patterns across loci can be readily summarized by comparing the levels of species divergence across loci. We computed the net nucleotide divergence *d*, which is the average divergence between *B. azoricus* and *B. puteoserpentis minus* the average sequence divergence within both species (π_puteo_+π_azo_)/2). Without inter-species gene flow, *d* should be positive, while under a model in which *B. azoricus* and *B. puteoserpentis* have exchanged genes since the separation from their common ancestry, *d* may fall down to zero and even become negative. The quantity *d* was positive for loci CO1, SAHH and close to zero for the remaining loci (Figure 4). *d* can then be compared to the divergence obtained between the two Atlantic species and their Pacific outgroup. In the absence of gene flow, a positive relationship is expected between evolutionary rates. However, the relationship between the *B. azoricus*/*B. puteoserpentis* divergence and the Pacific/Atlantic divergence was not significant. Five loci (SAHH, COL1, LYSO, GF1A, ITS2) out of the eight displayed similar ratios (Figure 5). Three genes seemed to depart from the genomic trend. CO1 and GF1B exhibited higher values, while EF1α displayed a lower ratio.

**Figure 6:**
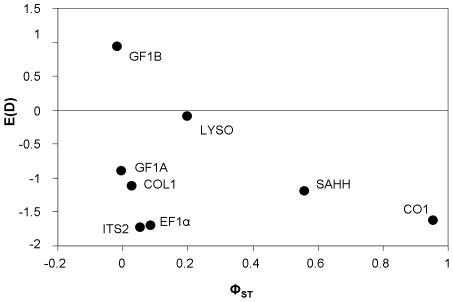
Average Tajima's D values as a function of the Φ_PhiST_.

Tajima's D tests [37] were used to measure the deviation from the mutation/drift equilibrium under neutrality (Table 2). In the three mussel populations, Tajima's *D* was mostly negative and sometimes significantly so (CO1, EF1α, ITS2). However, the LYSO locus displayed a D close to zero and the GF1B locus displayed a significantly positive D value. As population structure has an important impact on Tajima's D, the average D was plotted against Φ_ST_ in Figure 6. The higher Tajima's D observed at loci LYSO and GF1B could likely be the consequence of secondary introgression of divergent alleles while low gene flow at the CO1 and SAHH loci prevented divergent alleles to segregate within the same population. However, it remained puzzling why the D was so low at the remaining four loci. To complement Tajima's D tests, a multi locus HKA test has been performed. This test indicates that the ratio of polymorphism to divergence did not vary significantly among loci, however, the result for *B. puteoserpentis* was nearly significant: sum of deviation = 3.9587 (df = 7; *P* = 0.7845); 7.7511 (df = 7; *P* = 0.3550); 13.3811 (df = 7; *P* = 0.0633), for *B. azoricus* Rb vs *B. thermophilus* 7S; *B. azoricus* MG vs *B. thermophilus* 7S; and *B. puteoserpentis* vs *B. thermophilus* 7S respectively. We also computed the Ramos-Onsins and Rozas' *R_2_* statistic [38]. *R_2_* is a statistic based on the difference between the number of singletons per sequence and the average number of nucleotide differences and is especially well designed and powerfull to detect population expansion. *R_2_* was significant for CO1, COL1, (for all the populations), GF1B, ITS2 (for the *B. puteoserpentis*_Logatchev population), and SAHH (for the *B. azoricus*_Menez Gwen population).

The analysis of patterns of genetic diversity revealed a strong contrast among loci. Although some loci have well registered a history of divergence, exhibiting two distinct groups of alleles that were sometimes mixed up (e.g. GF1B) and sometimes not (e.g. CO1), other genes did not (e.g. COL1). Tajima's D was mostly negative suggesting population expansion, especially in *B. azoricus*. However, the homogenisation of divergent alleles was strong enough at loci GF1B and LYSO to restore positive D values. Possibly, selection could have superposed to history and demography and might explain that 3 genes (CO1, SAHH, and EF1α) departed from the averaged evolutionary rate estimated from the divergence with the Pacific species.

### Isolation-with-Migration model

**Figure 7:**
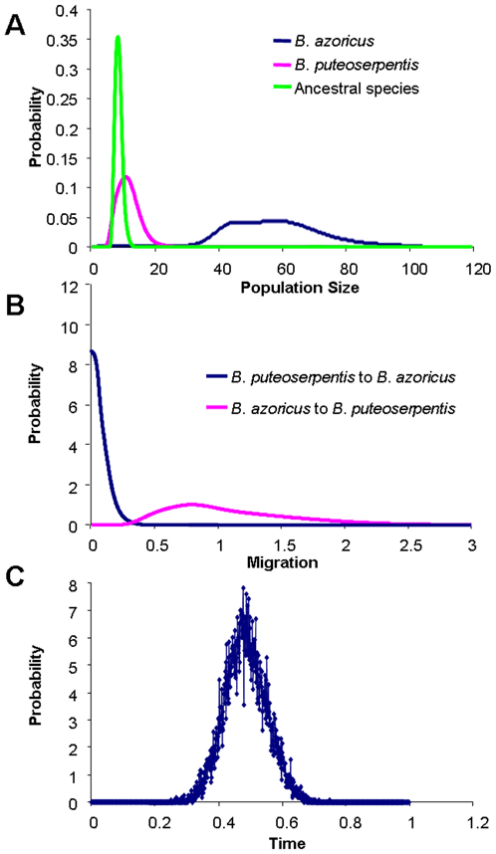
The marginal posterior probability distribution for model parameters (scaled by the neutral mutation rate). Curves are shown for the analysis with and for all *B. azoricus* and *B. puteoserpentis* (a) population size, (b) migration, (c) divergence time.

Repeated runs of the IMa program revealed unambiguous marginal posterior probability distributions of the parameters for the two actual species and the ancestral one. The peaks of the six parameter estimates were confined to fairly narrow ranges with corresponding posterior distribution illustrated in the Figure 7. In order to convert parameter estimates in more easily interpreted units, we estimated substitution rates for each locus from the divergence between the species of *B. thermophilus* from the EPR and its sister-species from 37°S, under the hypothesis that deep-sea species have been separated during the formation of the Eastern Island microplate (at 25°S) about 5.9 millions years ago [49]. The Eastern Island microplate was created on the axis of the ancestral Pacific ridge, and is thus responsible of the split of the South Pacific hydrothermal fauna north and south of this island. A geometric mean of the *B. thermophilus* EPR - 37°S DNA sequence divergence across loci was calculated and subsequently used to convert the time since separation parameter, *t*, to divergence in years. The mutation rate can be estimate as *r* = *D*/2*T*, where *D* is the pairwise sequence divergence and *T* is the time of divergence, multiplied by 2 to account for the age of each lineage. The geometric divergence for the mitochondrial and the seven nuclear genes is 1.07%. This divergence gave a substitution rate of 0.09% per MY. These values are in average twice as low as previous evolution rates estimated by Chevaldonné et al. [50] and Johnson et al. [51] for other hydrothermal invertebrates using the mtCO1 gene. However, this substitution rate is nearly equal to the rate of 0.10% per MY found by Johnson et al. [51] on a nuclear locus. Taking into account the length of each gene (Table 2), we estimated the number of mutations per locus per year to be from 0.25×10^−6^ (ITS2) to 2.07×10^−6^ (CO1). The geometric mean, u = 0.63×10^−6^ was used to rescale the IMa parameter estimates from the combined analysis. We used this value to directly convert the estimate *t* (the number of substitutions) to a number of years since population splitting.

To convert estimates of the population mutation rate parameters (θ_1_, θ_2_, and θ_A_) to estimates of effective population size (N_1_, N_2_, and N_A_, respectively), we estimated the generation time of *Bathymodiolus* from previous estimations of the mussel individual growth rate: 0.6 to 1.1 cm per year [52], [53] and the size at which individuals reach their sexual maturity (2 years: the minimum size observed for individuals able to reproduce is 19.7 mm for male and 31.9 mm for female (unpublished data from the BIOSPEEDO 2004 cruise). Calculations yielded an estimated number of mutations per generation per locus of 1.26×10^−6^.

Estimates of the effective population size are shown in Table 4. The estimate of the *B. azoricus* population size was five times greater than that of *B. puteoserpentis* and the ancestral one, which suggests population expansion in *B. azoricus*. The marginal posterior probability distribution of the divergence parameter, *t*, revealed a sharp peak at 0.477 (Figure 7), which converted into a divergence time of 0.758 MYA with the 90% HPD interval ranged from 0.599 to 0.953 MYA (Table 4). The multi-locus estimates of migration parameters revealed an asymmetric gene flow from *B. azoricus* to *B. puteoserpentis* with a mode around 2*Nm* = 4.3 (Figure 7, Table 4), while the reverse flow from *B. puteoserpentis* to *B. azoricus* did not appear to significantly depart from zero.

**Table 4:** Estimates and the 90% Highest Posterior Density (HPD) Intervals of Demographic Parameters from IMa multi locus analyse.

Comparison	θ1	θ2	θA	m1	m2	t	N1	N2	NA	M1 = 2N1m1	M2 = 2N2m2	t (years × 1000)
**Bazori × Bputeo**												
Estimate	57.936	10.829	8.224	0.001	0.792	0.477	11,497.577	2,149.008	1,632.117	0.01	4.29	758
lower 90% HPD	35.844	5.998	6.558	0.001	0.372	0.377	7,113.292	1,190.341	1,301.395			600
higher 90% HPD	78.225	16.545	10.326	0.165	1.827	0.600	15,523.971	3,283.425	2,049.147			953

**Figure 8:**
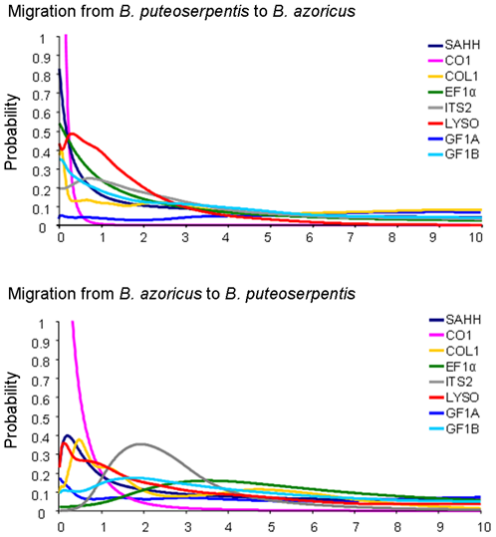
IMa single locus probability distributions of migration rates for each of the 8 loci.

Although the multi-locus estimates benefited from the statistical power of the full information contained in the eight loci the single locus analysis of population differentiation (Table 5) suggested that gene-flow could vary between genome regions as expected in the model of semi-permeable barriers to gene flow [7]. A single locus analysis of divergence with gene-flow under the Isolation with Migration model was therefore conducted. For this analysis we compared the outputs obtained with the IM [46] [23] and the IMa [45] programs. As results were very similar, we here present the results obtained with the IMa program that retrieved graphically clearer posterior distributions. Results are presented in Table 5 as a series of single-locus probabilities and in Figure 8 where posterior distributions are drawn. The mode, 90% HPDs and shapes of the posterior distributions allowed us to distinguish three categories of loci: (1) one locus without any evidence of migration (max at 0 in both direction), CO1; (2) four loci with a maximum of the marginal posterior distribution different from 0 at least in one direction but for which the hypothesis of no gene flow was not be rejected (lower 90% HPD = 0), SAHH, COL1, LYSO and GF1B; and (3) three loci with convincing evidence of gene flow in at least one direction (lower 90% HPD>0), EF1α, GF1A and ITS2. The flat, continuously increasing distribution observed at the GF1A locus and the absence of a clear peak explained that the maximum for m2 was inferred at the higher value of the parameter range computed (Table 5). Despite the lack of power of the single locus analyses, differences between extreme values showed up. For instance, loci ITS2 and EF1α showed distinguishable probability distribution to locus CO1 (Figure 8), with the former suggesting evidence of gene flow while not the latter. Estimates of the mean time of migration events have been obtained for the six loci with no-null migration, even if not significant (EF1α, SAHH, COL1, LYSO, GF1B and ITS2) and are provided in Table 5. This analysis suggested the existence of old migration events, which averaged at the half of the divergence period (mtime/t ratio in Table 5) around 0.4 MYA.

**Table 5:** Estimates and the 90% Highest Posterior Density (HPD) Intervals of Migration Rate Parameters, and maximum of the mean time of migration events (*mtime*), obtained from IMa analyses of the 8 loci.

Locus		m1 (from *B. puteoserpentis to B. azoricus*)	m2 (from *B. azoricus to B. puteoserpentis*)	t	t in years ×1000	mtime in year × 1000 (mtime/t)	
						m1	m2
CO1							
	Estimate	0.005	0.005	0.453	719	-	-
	lower 90% HPD	0.005	0.005	0.213			
	higher 90% HPD	0.195	1.275	4.998			
EF1α							
	Estimate	0.005	3.535	0.418	664	-	433 (0.65)
	lower 90% HPD	0.005	1.355	0.193			
	higher 90% HPD	6.965	9.115	4.998			
SAHH							
	Estimate	0.005	0.185	0.493	783	-	351 (0.45)
	lower 90% HPD	0.005	0.005	0.161			
	higher 90% HPD	8.025	8.295	1.999			
COL1							
	Estimate	0.005	0.465	1.087	1 726	-	814 (0.47)
	lower 90% HPD	0.005	0.005	0.738			
	higher 90% HPD	8.895	6.935	1.507			
LYSO							
	Estimate	0.185	0.005	0.317	503	210 (0.42)	-
	lower 90% HPD	0.005	0.005	0.119			
	higher 90% HPD	6.545	6.585	1.999			
GF1A							
	Estimate	0.007	14.992	0.787	1 249	-	No peak
	lower 90% HPD	0.007	1.987	0.333			
	higher 90% HPD	14.993	14.993	1.383			
GF1B							
	Estimate	0.005	1.715	0.617	980	-	663 (0.68)
	lower 90% HPD	0.005	0.005	0.077			
	higher 90% HPD	8.055	8.245	4.997			
ITS2							
	Estimate	0.705	1.785	1.318	2 092	1790 (0.86)	1473 (0.7)
	lower 90% HPD	0.005	0.575	0.872			
	higher 90% HPD	7.375	5.265	4.998			

### Evidence of a selective sweep at the SAHH locus

**Figure 9:**
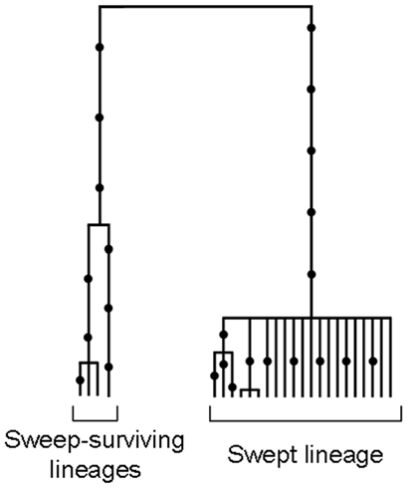
Reconstructed genealogy of the SAHH locus for *B. azoricus*. Mutations are indicated with dots.

SAHH is the only nuclear locus exhibiting a strong differentiation between the two species and a high *d*/*D_ATL/PAC_* ratio. The hypothesis that selection could have played a role in the evolution of this locus was therefore hypothesised. Although Tajima's D and HKA tests were not significant, the Fay and Wu's H test which is designed to be sensible to an excess of high frequency derived variants was significantly different from zero (H = −8.52, P = 0.04). The SAHH data were compatible with the infinite mutation model in *B. azoricus* and the single gene tree was reconstructed in Figure 9. The gene tree shows the partially star-shaped genealogy observed at this locus in *B. azoricus* which is characteristic of selective sweeps [54]. We therefore used the coalescence-based maximum-likelihood method of Galtier, Depaulis, and Barton [44]. Results of the runs indicated that the model with a selective sweep was significantly better supported than the model without diversity reducing event (θ = 13.2, S = 0.7, T = 0.22, Likelihood ratio test: P = 0.006).

### Evidence for secondary introgression from interclade recombinant alleles at the GF1B locus

**Figure 10:**
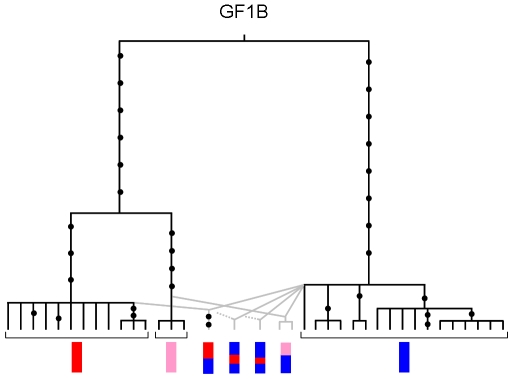
Ancestral recombination graph of the GF1B locus and schematic map of recombinant alleles. Mutations are indicated with dots. Dashed lines indicate that the parental allele of the recombinant could not be precisely identified.

The GF1B locus displayed positive Tajima's D values (Table 2), a fast rate of evolution in the Atlantic species when compared to the divergence from the outgroup *B. thermophilus* (Table 2), and evidence of recombination between divergent alleles. Although highly reticulated, a closer look at the phylogenetic network in Figure 3 revealed two groups of alleles and intermediate sequences with small terminal branches. One expects ancestral lineages to exhibit long terminal branches and these sequences seemed better explained by recombination. We conducted a detailed analysis of recombination the results of which are synthesized in the ancestral recombination graph presented in Figure 10. Four distinct recombination events between well distinct and divergent alleles have been identified. The interesting result of this analysis was that the diversity observed at this locus allowed to infer with little ambiguity that recombination between divergent alleles occurred recently after a long period during which recombination did not happened. This finding is in agreement with the hypothesis of secondary introgression of divergent alleles after a period of divergence in allopatry.

### Allele frequency spectra: searching for the footprint of interrupted gene-flow in the gene history

**Figure 11:**
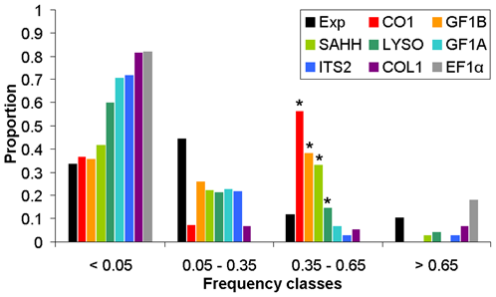
Distribution of the average frequency of the variant nucleotidic sites (when compared to the outgroup *B. thermophilus*) in the two Atlantic species. Stars indicate a significant excess of mutations with an intermediate frequency.

As the GF1B locus provided compelling evidence of secondary introgression, we searched for the footprint of interrupted gene flow at other loci. The idea was that intragenic recombination can erase the divergence produced by a period of allopatry as it was partly the case for the GF1B locus. Substitutions that were fixed during allopatry would then be dispatched among chromosomes by recombination but if introgression was balanced enough and the number of substitutions was high enough, an excess of mutations in intermediate frequency should be observed. In Figure 11 is presented the distribution of the average frequency of variant nucleotidic sites (when compared with the outgroup sequences) in the two Atlantic species. Logically, the CO1 and SAHH loci showed an excess of mutations with an intermediate average frequency which are indeed the fixed differences between the two species. The GF1B locus also displayed a clear excess of mutations with an intermediate average frequency which correspond to the substitutions of the internal branch in Figure 10. For the other five genes, the main effect was an excess of rare variants which can be explained by demography or a slightly deleterious effect of polymorphic mutations. However, once the excess of rare variants was accounted for, an excess of mutations with an intermediate frequency was still detected at the locus LYSO (Fisher's exact test: p = 0.01).

## Discussion

The two species of *Bathymodiolus* discovered along the Mid-Atlantic Ridge [55], [56] were first described from their morphologies and considered to be distinct species named *B. azoricus* and *B. puteoserpentis* [57]. O'Mullan et al. [19] and Won et al. [20], however, reported that these two species were able to hybridise at the Broken Spur vent field (29°10′N/43°10′W; 3350 m depth) situated between the two species' ranges. These authors have interpreted local hybridisation between the two Atlantic species as the result of an accidental secondary contact between two divergent species [20] which is the traditional interpretation in the hybrid zone framework [58]. However, it is also well-known in the hybrid zone literature that it is extremely difficult to tell apart secondary contact zone from parapatric primary differentiation [59] and the former hypothesis was preferred because the latter was thought more unlikely to happen. Recently, a new burst of data relevant to this question lead to a re-evaluation of the easiness of speciation with gene flow [5]. In the case of *B. azoricus* and *B. puteoserpentis*, the geographic context is not easily reconcilable with the opportunity for a secondary contact as the Northern Mid-Atlantic Ridge is a dead-end limited to the north by Iceland. This is why our eight-locus analysis of DNA sequence polymorphism was aimed to reconstruct the history of divergence and gene flow between these two closely-related organisms in the original environment of hydrothermal vents, in an to attempt to accumulate evidence for one of the two scenarios, secondary contact or parapatric speciation.

### Inferences from the isolation with gene-flow model

As recently done for an increasing number of pairs of closely related species [2], [4], [5], the multi-locus fit to an isolation with gene flow model produced useful inferences to understand how isolation happened between *B. azoricus* and *B. puteoserpentis*. First, divergence was estimated to date back to more than half a million years. Secondly, we estimated that the effective population size of *B. azoricus* was 2–5 times greater than that of *B. puteoserpentis* and the ancestral population suggesting population expansions in *B. azoricus*. This finding is in agreement with census population sizes observed for both species –dense mussel beds in the case of *B. azoricus* [60] *versus* scattered individuals in the case of *B. puteoserpentis* [61]. Finally, evidence for gene-flow was unambiguous although mainly asymmetrical from the *B. azoricus* to *B. puteoserpentis*. The asymmetry of gene flow can have several explanations. (i) Hybrid zone theory predicts asymmetry of a genetic barrier to gene flow according to the density of populations [62]. Both population genetics inferences and observations in the field are in accordance for a denser and bigger population of *B. azoricus*, which would agree well with the asymmetry of the barrier to gene flow. (ii) Hybrid zone theory also predicts that moving hybrid zones are expected to leave a footprint in the form of asymmetrical introgression [58], [63]. The inference of an asymmetric gene flow could therefore also be in accordance with a northward displacement of the hybrid zone. A recent review [63] suggests that hybrid zone movements could be more frequent than previously thought. The direction of introgression observed here would be in accordance with a northward displacement of the *Bathymodiolus* hybrid zone. Providing a displacement of the zone was indeed the explanation of asymmetric introgression, one might hypothesised it could have been favoured by abyssal currents along the Mid-Atlantic Ridge. Indeed, the Brazil basin represents a vortex zone, from which the AABW (Antarctic Bottom Water) is subdivided into deep-sea water jets that move northward against the western flank of the MAR to reach the Florida Escarpment [64] and, enters the Mid Atlantic Ridge using the Vema Fracture Zone located near 10°N/MAR. At this point, the residual jet favours larval dispersal further north [65]. (iii) Finally, asymmetric gene flow could be a consequence of an asymmetry of hybridisation, either genome-wide or depending on the isolation genes principally responsible for the barrier to gene-flow generated on the chromosomal region a locus belongs to. Distinguishing among these various explanations however is difficult and would require experiments in the lab hardly achievable with *Bathymodiolus* mussels.

The history of gene-flow: what evidence for a period of no gene-flow?

When we undertook the analysis of DNA sequences in *Bathymodiolus* we had in mind that we might be able to distinguish secondary contact from parapatric differentiation. However, it was not that simple and although evidence of gene flow was straightforward, it was unclear whether it meant genes were continuously exchanged during the whole divergence period or whether it remained compatible with a period without gene flow before secondary contact. Taking at face value, the result from the posterior distribution of the mean time of migration events could suggest migration mainly occurred around 0.4 MYA, half of the divergence period (Table 5). However, the statistical behaviour of this parameter has not been extensively studied and it is difficult to understand its true meaning. For instance, our data are enriched in rare variants (*e.g.* singletons) which can be explained in various ways (demography, selection, …) that are not compatible with the IM model assumptions and which could therefore result in an absence of gene flow in the recent past under the IM framework. The divergence being short relative to the coalescence time, this recent period freed from coalescence events, and therefore from migration events, in gene trees can contribute to artificially overestimate the mean time of migration events. It is unclear also how this parameter would react when the true story implies several periods of gene flow interrupted by periods of isolation. As the parameter is the mean time of all the migration events, the multimodality should not be detected. One might look more closely at the branching of easily identified introgressed alleles in observed gene trees in order to verify whether observed introgression times concur with the posterior estimates from a predefined model. Introgressed alleles are visible only when divergence is strong enough and gene flow low enough. This was the case for locus SAHH which exhibited fairly clear examples of introgression of *B. azoricus* alleles in the *B. puteoserpentis* background. We would like here to emphasise that this observation cannot be the result of the sampling of rare *B. azoricus* individual migrants in a *B. puteoserpentis* population because we sampled far away from the identified hybrid zone [19] and none of the introgressed individuals displayed a hybrid signature. We identified two alleles which are likely results of introgression at the SAHH locus, (see the two arrays in Figure 3), one (labelled 1 in Figure 3) coalesced at the tip of the network and was in accordance with recent secondary introgression while the other (labelled 2 in Figure 3) coalesced more deeply and could well indicate ancient gene flow. To a lesser extent, locus LYSO also exhibited strong divergence together with restricted gene flow and both kinds of branching were also observed (Figure 3).

Another useful information, although less studied (but see [66]), is intragenic recombination, because recombination can only occur between alleles that are segregating together within the same populations and genomes. The reconstructed ancestral recombination graph of the GF1B locus (Figure 10) allowed us to identify the presence of recent recombinant alleles between two divergent clades of alleles. The recent take again of recombination at the GF1B locus is a strong argument for the hypothesis of secondary introgression. However, it also suggests that secondary introgression operates since a sufficiently long period of time to allow newly formed recombinants to reach a high enough frequency to be sampled. According to Figure 10, recombination acted during roughly the 10–20% end of the global divergence period which would result in a guesstimate of the time of the secondary contact of 50–200 thousands of years (from the estimate of the divergence time in Table 4). This guesstimate is not in accordance with the mean time of migration events estimated with IMa. The true value is probably in-between. The important point is the relevant evidence for the existence of a period without gene flow that prevented recombination.

Secondary contact and subsequent introgression would therefore explain our results. Because no species can enter the Atlantic ridge further north of Iceland, the only scenario of the MAR colonisation that allows the formation of a secondary contact zone is a first colonisation by the species *B. azoricus*. Its geographic distribution was then challenged by *B. puteoserpentis* located further south. Such a scenario implies that the Mid Atlantic Ridge would have been independently colonised twice. As both vent species seem to be related to the *B. boomerang* complex of species (sister group) located at cold seeps from the deep-sea Caribbean region [17], geographic isolates originated from either Barbados accretion prism or the Florida escarpment were likely to colonise the rift separately and met subsequently to hybridise.

### The impact of selection on the differentiation process

The effect of a possible selective sweep at the most discriminating nuclear locus SAHH has been detected. In addition, negative and significant values of the Tajima's D and Ramos-Onsins and Rozas's *R_2_* were found at the mitochondrial gene CO1 which remains the only locus differentially fixed between the two species. Recurrent selective sweeps are indeed likely to rejuvenate the coalescence time of the mitochondrial genome in species with big populations such as marine molluscs [67]. One can therefore argue that selection helped to accelerate the evolution toward monophyly at these two loci, explaining their status of (semi-) diagnostic markers. However, at least two other loci, LYSO and GF1B, also registered a strong divergence between two groups of alleles that are interpreted to have introgressed secondarily. Therefore, SAHH and CO1 also need to be in genomic regions influenced by isolation genes responsible for the genetic barrier to gene-flow. One can then hypothesise that the sweeps detected were the consequence of positive selection at speciation genes linked to SAHH and CO1 [68]. Some other ecological elements like the symbiont composition of the *Bathymodiolus* gills can also act as selective constraints. Both species indeed yield two kinds of symbionts, one being a sulfo-oxidizing bacteria and the other a methanogen coccus. Both symbionts co-exist in the mussel gills [69], [70] but can be easily lost depending on the fluid composition [71] or selectively acquired by each species in the environment [72].

Another possible effect of selection we already discussed in a previous paper is slight purifying selection on polymorphic mutations. We suspected such a deleterious effect of polymorphic mutations at the EF1α locus in *Bathymodiolus* [26]. Purifying selection at some locus can inflate the contrast observed between loci with some having registered well the period of allopatry with multiple fixations (e.g. CO1, SAHH, LYSO) while other loci having evolved too slowly and exhibiting an excess of rare, slightly deleterious, variants (e.g. EF1α, ITS2; see Figure 11). However, despite low evolutionary rates, the species structure was still observable and alleles segregate into two distinct groups. For some other loci (COL1, GF1A), the situation seemed more complex. Other confounding effects can be a high rate of intragenic recombination that artificially makes younger the divergence [73] such as observed at GF1A, or the complete replacement of one lineage by introgression. Such processes were reported in *Drosophila* to explain complex genealogies between *D. permilis*, *D. pseudoobscura* and *D. p. bogotana*. [74]; [75].

## 

### Conclusion

The two closely-related species of *Bathymodiolus* from the Mid-Atlantic Ridge were known to hybridise locally within the narrow hybrid zone of Broken Spur [19], [20]. The analysis of populations located at both extremities of the mussel range by using a multilocus approach indicated that the divergence started more than half a million years ago and that gene flow occurs or has occurred, although mainly asymmetrically from *B. azoricus* to *B. puteoserpentis*. Finally, we obtained some evidences that gene-flow was due to secondary introgression after a period of allopatry. The main argument is the recent restart of intragenic recombination after a period where it proved to be blocked at the GF1B locus.
